# 1,3,5,7-Tetra­kis(4-iodo­phen­yl)adamantane benzene tetra­solvate

**DOI:** 10.1107/S1600536810021744

**Published:** 2010-06-16

**Authors:** Jan W. Bats, Steffen Pospiech, Thomas F. Prisner

**Affiliations:** a Institut für Organische Chemie, Universität Frankfurt, Max-von-Laue-Strasse 7, D-60438 Frankfurt am Main, Germany; b Institut für Physikalische und Theoretische Chemie, Universität Frankfurt, Max-von-Laue-Strasse 7, D-60438 Frankfurt am Main, Germany

## Abstract

The title mol­ecule, C_34_H_28_I_4_·4C_6_H_6_, has crystallographic 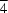 symmetry and crystallizes with four symmetry-related benzene solvent mol­ecules. The phenyl group is eclipsed with one of the adamantane C—C bonds. The tetra­phenyl­adamantane units and the benzene solvent mol­ecules are connected by weak inter­molecular phen­yl–benzene C—H⋯π and benzene–benzene C—H⋯π inter­actions. In the crystal, mol­ecules are linked along the *c*-axis direction *via* the iodo­phenyl groups by a combination of weak inter­molecular I⋯I [3.944 (1) Å] and I⋯π(phen­yl) [3.608 (6) and 3.692 (5) Å] inter­actions.

## Related literature

For the preparation of the title compound, see: Li *et al.* (2002 ▶). For the crystal structure of a related compound, see: Boldog *et al.* (2009 ▶). For inter­molecular inter­actions of I atoms, see: Pedireddi *et al.* (1994 ▶); Thaimattam *et al.* (1998 ▶)
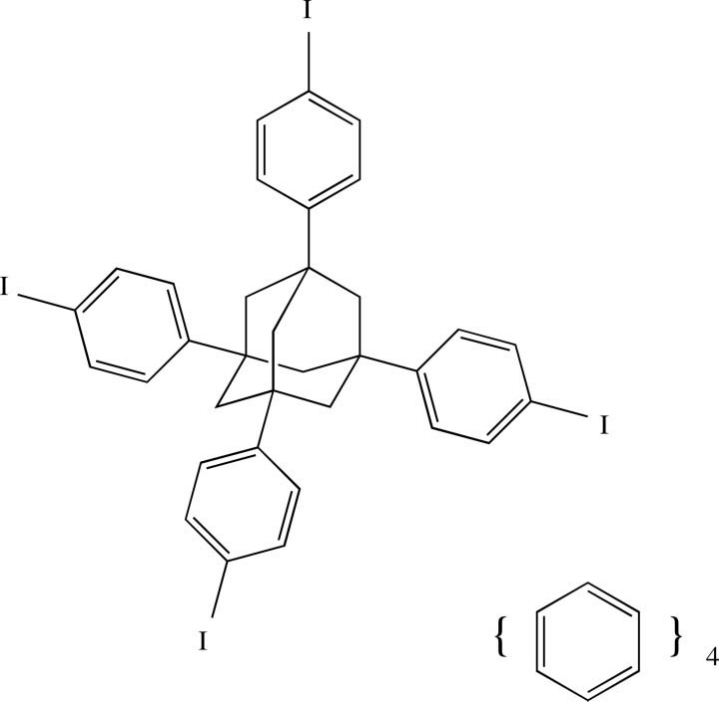


## Experimental

### 

#### Crystal data

C_34_H_28_I_4_·4C_6_H_6_
*M* *_r_* = 1256.60
Tetragonal, 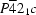

*a* = 18.883 (3) Å
*c* = 7.2442 (19) Å
*V* = 2583.1 (9) Å^3^
*Z* = 2
Mo *K*α radiation
μ = 2.45 mm^−1^
*T* = 164 K
0.60 × 0.20 × 0.12 mm

#### Data collection

Siemens SMART 1K CCD diffractometer
Absorption correction: multi-scan (*SADABS*; Sheldrick, 2000 ▶) *T*_min_ = 0.509, *T*_max_ = 0.751
35313 measured reflections
2953 independent reflections
2365 reflections with *I* > 2σ(*I*)
*R*_int_ = 0.063

#### Refinement

*R*[*F* ^2^ > 2σ(*F* ^2^)] = 0.040
*wR*(*F* ^2^) = 0.105
*S* = 1.05
2953 reflections
141 parameters
H-atom parameters constrained
Δρ_max_ = 1.76 e Å^−3^
Δρ_min_ = −0.82 e Å^−3^
Absolute structure: Flack (1983 ▶), 1263 Friedel pairs
Flack parameter: −0.01 (4)

### 

Data collection: *SMART* (Siemens, 1995 ▶); cell refinement: *SMART*; data reduction: *SAINT* (Siemens, 1995 ▶); program(s) used to solve structure: *SHELXS97* (Sheldrick, 2008 ▶); program(s) used to refine structure: *SHELXL97* (Sheldrick, 2008 ▶); molecular graphics: *SHELXTL* (Sheldrick, 2008 ▶); software used to prepare material for publication: *SHELXL97*.

## Supplementary Material

Crystal structure: contains datablocks global, I. DOI: 10.1107/S1600536810021744/lh5063sup1.cif

Structure factors: contains datablocks I. DOI: 10.1107/S1600536810021744/lh5063Isup2.hkl

Additional supplementary materials:  crystallographic information; 3D view; checkCIF report

## Figures and Tables

**Table 1:** Hydrogen-bond geometry (Å, °) *Cg*1 and *Cg*2 represent the midpoint of the C13—C14 bond and the centroid of the C10–C15 ring, respectively.

*D*—H⋯*A*	*D*—H	H⋯*A*	*D*⋯*A*	*D*—H⋯*A*
C5—H5*A*⋯*Cg*1^i^	0.95	2.91	3.833 (9)	163
C10—H10*A*⋯*Cg*2^ii^	0.95	2.85	3.733 (9)	156

Symmetry codes: (i) 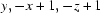; (ii) 
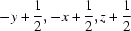
.

**Fig. 1.**
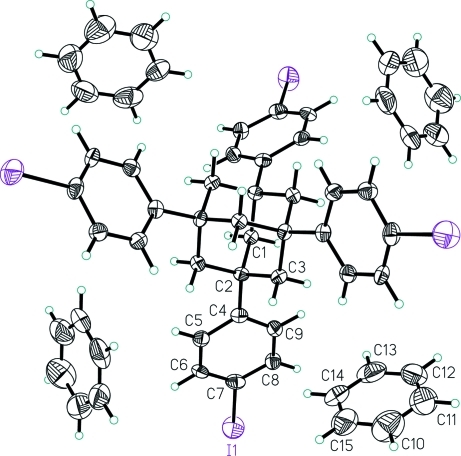
The structure of the title compound shown with 50% probability displacement ellipsoids. The H atoms are drawn as small spheres of arbitrary radius. Unlabelled atoms are related to labelled atoms by 4 symmetry.

**Fig. 2.**
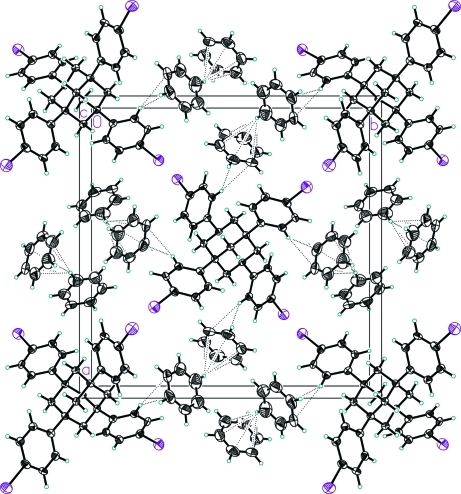
The crystal packing of the title compound, viewed along the c axis. C—H···π contacts are shown as dotted lines.

**Fig. 3.**
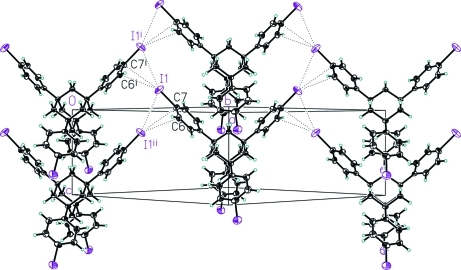
Section of the crystal structure showing the linking of the 1,3,5,7-tetrakis(4-iodophenyl)adamantane molecules by intermolecular contacts between the iodophenyl groups. The symmetry codes are i: 1/2 - y, 1/2 - x, -1/2 + z and ii: 1/2 - y, 1/2 - x, 1/2 + z.

## supplementary crystallographic information

### Comment

The title compound was prepared as a precursor for the synthesis of EPR-active tetrahedral model systems.

The asymmetric unit contains a quarter of a 1,3,5,7-tetrakis(4-iodophenyl)adamantane molecule and one benzene solvent molecule. The molecular structure is shown in Fig. 1. The substituted adamantane molecule has 4 symmetry. The conformation of the tetraphenyladamantane unit is very similar to the conformation in the crystal structure of 1,3,5,7-tetraphenyladamantane (Boldog *et al.*, 2009), with the phenyl group eclipsed with one of the adamantane C—C bonds (torsion angle C5—C4—C2—C3^i^ = 5.7 (6)° [symmetry i: *y*, 1 - *x*, 1 - *z*]).

The crystal packing is shown in Fig. 2. The tetraphenyladamantane and the benzene solvent molecules are connected by intermolecular C—H···π interactions (Table 1, *Cg*1 and *Cg*2 represent the midpoint of the C13—C14 bond and the centroid of the C10—C15 ring respectively). There is a C—H···π contact between the phenyl ring and a benzene solvent molecule [angle between planes of rings: 72.9 (2)°]. The C_phenyl_—H bond does not point to the center of the benzene ring, but closer to the midpoint of the C13—C14 bond. The benzene solvent molecules are connected along the c direction by an additional C—H···π contact. The angle between the planes of the donor and acceptor benzene molecules is 83.3 (2)° and the donor C—H bond points closely to the center of the acceptor ring.

Each C—I bond points to the iodophenyl group of a neighboring molecule as shown in Fig. 3. The shortest contact distances are: I1···I1^i^ = 3.944 (1) Å, I1···C6^i^ = 3.608 (6)Å and I1···C7^i^ = 3.692 (5)Å (symmetry i: 1/2 - *y*, 1/2 - *x*, -1/2 + *z*). The angles for these contacts are: C7—I1···I1^i^ = 158.6 (1)°, C7—I1···C6^i^ = 153.9 (2)° and C7—I1···C7^i^ = 167.0 (2)°. This combination of weak interactions link the 1,3,5,7-tetrakis(4-iodophenyl)adamantane molecules along the c direction. The significance of these interactions for crystal packing has been discussed by Pedireddi *et al.* (1994) and Thaimattam *et al.* (1998).

### Experimental

The title compound was prepared as described by Li *et al.* (2002). Single crystals were obtained by recrystallization of the compound from benzene. The crystals rapidly decomposed in the air at room temperature. Therefore a crystal was taken from the mother liquor and was rapidly cooled to 164 K.

### Refinement

The H atoms were positioned geometrically and treated as riding: C_non-planar_—H=0.99 Å, C_planar_—H=0.95Å and *U*_iso_(H)=1.2*U*_eq_(C). The absolute structure was determined using 1263 Friedel pairs.

### Crystal data

**Table:**

C_34_H_28_I_4_·4C_6_H_6_	*D*_x_ = 1.616 Mg m^−^^3^
*M**_r_* = 1256.60	Mo *K*α radiation, λ = 0.71073 Å
Tetragonal, *P*42_1_*c*	Cell parameters from 103 reflections
Hall symbol: P -4 2n	θ = 3–23°
*a* = 18.883 (3) Å	µ = 2.45 mm^−^^1^
*c* = 7.2442 (19) Å	*T* = 164 K
*V* = 2583.1 (9) Å^3^	Rod, colorless
*Z* = 2	0.60 × 0.20 × 0.12 mm
*F*(000) = 1224	

### Data collection

**Table:**

Siemens SMART 1K CCD diffractometer	2953 independent reflections
Radiation source: normal-focus sealed tube	2365 reflections with *I* > 2σ(*I*)
graphite	*R*_int_ = 0.063
ω scans	θ_max_ = 27.5°, θ_min_ = 1.5°
Absorption correction: multi-scan (*SADABS*; Sheldrick, 2000)	*h* = −24→24
*T*_min_ = 0.509, *T*_max_ = 0.751	*k* = −24→24
35313 measured reflections	*l* = −9→9

### Refinement

**Table:**

Refinement on *F*^2^	Secondary atom site location: difference Fourier map
Least-squares matrix: full	Hydrogen site location: inferred from neighbouring sites
*R*[*F*^2^ > 2σ(*F*^2^)] = 0.040	H-atom parameters constrained
*wR*(*F*^2^) = 0.105	*w* = 1/[σ^2^(*F*_o_^2^) + (0.06*P*)^2^] where *P* = (*F*_o_^2^ + 2*F*_c_^2^)/3
*S* = 1.05	(Δ/σ)_max_ = 0.001
2953 reflections	Δρ_max_ = 1.76 e Å^−^^3^
141 parameters	Δρ_min_ = −0.82 e Å^−^^3^
0 restraints	Absolute structure: Flack (1983), 1263 Friedel pairs
Primary atom site location: structure-invariant direct methods	Flack parameter: −0.01 (4)

### Special details

**Table:**

Geometry. All e.s.d.'s (except the e.s.d. in the dihedral angle between two l.s. planes) are estimated using the full covariance matrix. The cell e.s.d.'s are taken into account individually in the estimation of e.s.d.'s in distances, angles and torsion angles; correlations between e.s.d.'s in cell parameters are only used when they are defined by crystal symmetry. An approximate (isotropic) treatment of cell e.s.d.'s is used for estimating e.s.d.'s involving l.s. planes.
Refinement. Refinement of *F*^2^ against ALL reflections. The weighted *R*-factor *wR* and goodness of fit *S* are based on *F*^2^, conventional *R*-factors *R* are based on *F*, with *F* set to zero for negative *F*^2^. The threshold expression of *F*^2^ > σ(*F*^2^) is used only for calculating *R*-factors(gt) *etc*. and is not relevant to the choice of reflections for refinement. *R*-factors based on *F*^2^ are statistically about twice as large as those based on *F*, and *R*- factors based on ALL data will be even larger.

### Fractional atomic coordinates and isotropic or equivalent isotropic displacement parameters (Å^2^)

**Table:**

	*x*	*y*	*z*	*U*_iso_*/*U*_eq_	Occ. (<1)
I1	0.25295 (2)	0.30544 (2)	−0.23303 (6)	0.05317 (17)	
C1	0.5000	0.5000	0.2564 (9)	0.0199 (12)	
H1A	0.5258	0.4664	0.1760	0.024*	0.50
H1B	0.4742	0.5336	0.1760	0.024*	0.50
C2	0.4466 (2)	0.4589 (2)	0.3747 (6)	0.0195 (9)	
C3	0.4875 (3)	0.4074 (2)	0.5016 (6)	0.0209 (9)	
H3A	0.4534	0.3815	0.5804	0.025*	
H3B	0.5129	0.3723	0.4248	0.025*	
C4	0.3977 (2)	0.4186 (2)	0.2437 (6)	0.0222 (8)	
C5	0.3250 (2)	0.4344 (2)	0.2272 (7)	0.0274 (10)	
H5A	0.3039	0.4677	0.3087	0.033*	
C6	0.2844 (3)	0.4020 (3)	0.0937 (6)	0.0287 (11)	
H6A	0.2354	0.4131	0.0842	0.034*	
C7	0.3139 (3)	0.3534 (3)	−0.0265 (6)	0.0310 (11)	
C8	0.3848 (3)	0.3345 (3)	−0.0094 (7)	0.0282 (11)	
H8A	0.4052	0.3000	−0.0885	0.034*	
C9	0.4250 (3)	0.3675 (3)	0.1261 (6)	0.0279 (10)	
H9A	0.4734	0.3544	0.1388	0.034*	
C10	0.4526 (5)	0.1182 (4)	0.7048 (12)	0.083 (2)	
H10A	0.4384	0.0903	0.8073	0.099*	
C11	0.5184 (5)	0.1096 (5)	0.6257 (15)	0.081 (3)	
H11A	0.5501	0.0748	0.6719	0.097*	
C12	0.5372 (4)	0.1500 (4)	0.4852 (13)	0.072 (2)	
H12A	0.5831	0.1449	0.4335	0.086*	
C13	0.4918 (4)	0.1992 (4)	0.4127 (10)	0.066 (2)	
H13A	0.5055	0.2268	0.3089	0.080*	
C14	0.4265 (4)	0.2084 (4)	0.4909 (11)	0.0612 (19)	
H14A	0.3947	0.2432	0.4445	0.073*	
C15	0.4085 (5)	0.1681 (4)	0.6319 (14)	0.078 (3)	
H15A	0.3628	0.1740	0.6849	0.094*	

### Atomic displacement parameters (Å^2^)

**Table:**

	*U*^11^	*U*^22^	*U*^33^	*U*^12^	*U*^13^	*U*^23^
I1	0.0530 (2)	0.0577 (3)	0.0488 (2)	0.00188 (19)	−0.0243 (2)	−0.0168 (2)
C1	0.023 (3)	0.022 (3)	0.015 (3)	0.000 (2)	0.000	0.000
C2	0.021 (2)	0.023 (2)	0.015 (2)	−0.0026 (18)	−0.0018 (17)	−0.0024 (17)
C3	0.026 (2)	0.020 (2)	0.017 (2)	−0.001 (2)	−0.0010 (18)	−0.0001 (17)
C4	0.026 (2)	0.026 (2)	0.015 (2)	−0.0043 (16)	0.0018 (19)	0.0027 (19)
C5	0.029 (2)	0.024 (2)	0.029 (2)	−0.0014 (17)	0.006 (2)	0.005 (2)
C6	0.019 (2)	0.031 (3)	0.036 (3)	−0.006 (2)	−0.002 (2)	0.000 (2)
C7	0.031 (3)	0.035 (3)	0.027 (2)	−0.008 (2)	−0.009 (2)	0.001 (2)
C8	0.030 (3)	0.029 (3)	0.026 (2)	0.002 (2)	−0.002 (2)	−0.004 (2)
C9	0.024 (2)	0.028 (3)	0.032 (2)	0.001 (2)	−0.002 (2)	−0.002 (2)
C10	0.105 (7)	0.070 (5)	0.073 (6)	−0.003 (5)	0.014 (5)	0.010 (4)
C11	0.071 (5)	0.066 (5)	0.106 (7)	−0.001 (5)	−0.030 (5)	0.012 (5)
C12	0.045 (4)	0.062 (5)	0.109 (7)	−0.011 (4)	0.013 (4)	−0.020 (5)
C13	0.080 (5)	0.045 (4)	0.074 (5)	−0.020 (4)	0.013 (4)	−0.020 (4)
C14	0.065 (5)	0.029 (4)	0.089 (5)	−0.007 (3)	−0.004 (4)	−0.008 (3)
C15	0.074 (6)	0.053 (5)	0.108 (7)	0.000 (4)	0.018 (5)	−0.027 (5)

### Geometric parameters (Å, °)

**Table:**

I1—C7	2.093 (5)	C7—C8	1.391 (7)
C1—C2^i^	1.535 (5)	C8—C9	1.388 (6)
C1—C2	1.535 (5)	C8—H8A	0.9500
C1—H1A	0.9900	C9—H9A	0.9500
C1—H1B	0.9900	C10—C15	1.364 (11)
C2—C4	1.528 (6)	C10—C11	1.378 (12)
C2—C3^ii^	1.540 (6)	C10—H10A	0.9500
C2—C3	1.545 (6)	C11—C12	1.321 (12)
C3—C2^iii^	1.540 (6)	C11—H11A	0.9500
C3—H3A	0.9900	C12—C13	1.369 (11)
C3—H3B	0.9900	C12—H12A	0.9500
C4—C9	1.387 (6)	C13—C14	1.368 (10)
C4—C5	1.409 (6)	C13—H13A	0.9500
C5—C6	1.378 (6)	C14—C15	1.319 (11)
C5—H5A	0.9500	C14—H14A	0.9500
C6—C7	1.382 (7)	C15—H15A	0.9500
C6—H6A	0.9500		
			
C2^i^—C1—C2	112.1 (5)	C6—C7—C8	120.1 (4)
C2^i^—C1—H1A	109.2	C6—C7—I1	121.1 (4)
C2—C1—H1A	109.2	C8—C7—I1	118.8 (4)
C2^i^—C1—H1B	109.2	C9—C8—C7	118.4 (4)
C2—C1—H1B	109.2	C9—C8—H8A	120.8
H1A—C1—H1B	107.9	C7—C8—H8A	120.8
C4—C2—C1	107.6 (3)	C4—C9—C8	122.8 (4)
C4—C2—C3^ii^	113.5 (4)	C4—C9—H9A	118.6
C1—C2—C3^ii^	107.9 (3)	C8—C9—H9A	118.6
C4—C2—C3	111.0 (4)	C15—C10—C11	118.1 (8)
C1—C2—C3	108.7 (3)	C15—C10—H10A	120.9
C3^ii^—C2—C3	107.9 (3)	C11—C10—H10A	120.9
C2^iii^—C3—C2	111.9 (4)	C12—C11—C10	119.6 (9)
C2^iii^—C3—H3A	109.2	C12—C11—H11A	120.2
C2—C3—H3A	109.2	C10—C11—H11A	120.2
C2^iii^—C3—H3B	109.2	C11—C12—C13	121.3 (8)
C2—C3—H3B	109.2	C11—C12—H12A	119.3
H3A—C3—H3B	107.9	C13—C12—H12A	119.3
C9—C4—C5	117.3 (4)	C14—C13—C12	119.5 (8)
C9—C4—C2	120.2 (4)	C14—C13—H13A	120.3
C5—C4—C2	122.4 (4)	C12—C13—H13A	120.3
C6—C5—C4	120.5 (4)	C15—C14—C13	118.7 (8)
C6—C5—H5A	119.7	C15—C14—H14A	120.7
C4—C5—H5A	119.7	C13—C14—H14A	120.7
C5—C6—C7	120.8 (5)	C14—C15—C10	122.8 (8)
C5—C6—H6A	119.6	C14—C15—H15A	118.6
C7—C6—H6A	119.6	C10—C15—H15A	118.6
			
C2^i^—C1—C2—C4	−178.4 (4)	C4—C5—C6—C7	0.0 (7)
C2^i^—C1—C2—C3^ii^	58.7 (3)	C5—C6—C7—C8	2.5 (7)
C2^i^—C1—C2—C3	−58.1 (3)	C5—C6—C7—I1	−178.8 (3)
C4—C2—C3—C2^iii^	175.9 (4)	C6—C7—C8—C9	−2.3 (7)
C1—C2—C3—C2^iii^	57.7 (5)	I1—C7—C8—C9	179.0 (3)
C3^ii^—C2—C3—C2^iii^	−59.1 (3)	C5—C4—C9—C8	2.9 (7)
C1—C2—C4—C9	61.9 (5)	C2—C4—C9—C8	−172.9 (4)
C3^ii^—C2—C4—C9	−178.8 (4)	C7—C8—C9—C4	−0.5 (7)
C3—C2—C4—C9	−57.0 (5)	C15—C10—C11—C12	1.1 (14)
C1—C2—C4—C5	−113.7 (4)	C10—C11—C12—C13	−1.9 (13)
C3^ii^—C2—C4—C5	5.7 (6)	C11—C12—C13—C14	2.2 (12)
C3—C2—C4—C5	127.4 (4)	C12—C13—C14—C15	−1.7 (11)
C9—C4—C5—C6	−2.6 (6)	C13—C14—C15—C10	0.9 (12)
C2—C4—C5—C6	173.1 (4)	C11—C10—C15—C14	−0.6 (13)

Symmetry codes: (i) −*x*+1, −*y*+1, *z*; (ii) *y*, −*x*+1, −*z*+1; (iii) −*y*+1, *x*, −*z*+1.

### Hydrogen-bond geometry (Å, °)

**Table:**

*Cg*1 and *Cg*2 represent the midpoint of the C13—C14 bond and the centroid of the C10–C15 ring, respectively.

**Table:**

*D*—H···*A*	*D*—H	H···*A*	*D*···*A*	*D*—H···*A*
C5—H5A···Cg1^ii^	0.95	2.91	3.833 (9)	163
C10—H10A···Cg2^iv^	0.95	2.85	3.733 (9)	156

Symmetry codes: (ii) *y*, −*x*+1, −*z*+1; (iv) −*y*+1/2, −*x*+1/2, *z*+1/2.
